# Management of Interstitial Ectopic Pregnancy in a Bicornuate Uterus Simulating an Incomplete Abortion

**DOI:** 10.7759/cureus.58351

**Published:** 2024-04-15

**Authors:** Dionysios G Galatis, Christos Benekos, Konstantina Kalaitzi, Panagiotis-Konstantinos Karachalios, Ioannis Chatzipanagiotis, Ippokratis Diamantakis, Foteini Anifantaki, Argyrios Monastiriotis, Vasileios Batsakoutsas, Nikolaos Kiriakopoulos

**Affiliations:** 1 Department of Obstetrics and Gynecology, National and Kapodistrian University of Athens School of Medicine, Athens, GRC; 2 V’ Department of Obstetrics/Gynecology, Helena Venizelou General and Maternity Hospital, Athens, GRC

**Keywords:** uterine rupture, interstitial pregnancy, congenital uterine anomalies, ectopic pregnancy, bicornuate uterus

## Abstract

The presentation of a bicornuate uterus may include miscarriages and menstrual abnormalities. The diagnosis could be in an incident of caesarean delivery, miscarriage or hysteroscopy. The possibility of misdiagnosis to an ectopic pregnancy is real. There are sonographical similarities between a pregnant horn of a bicornuate uterus and an ectopic pregnancy. We present in this article a case of interstitial pregnancy in a woman with a bicornuate uterus simulating symptoms of miscarriage. Congenital abnormalities necessitate the availability of the best diagnostic tools at the disposal of the medical practitioners. Ultrasound scan is an important aid for practitioners to choose the best therapeutic approach.

## Introduction

Congenital anomalies of the uterus present an increased chance of endometriosis, hematometra, malformations of the urinary tract and difficulties in conceiving. The congenital malformation known as bicornuate uterus has a rate of occurrence at 0.4% [1]. A bicornuate uterus is a lateral fusion defect that is known as a type III uterine anomaly under the American Society for Reproductive Medicine classification [2]. The possibility of cervical insufficiency, spontaneous abortion, uterine rupture and preterm delivery is increased in a bicornuate uterus [1].

The presentation of a bicornuate uterus may include miscarriages and menstrual abnormalities or be entirely without symptoms. The diagnosis could be in an incident of caesarean delivery, miscarriage or hysteroscopy. The probability of misdiagnosis to an ectopic pregnancy is real. There are sonographical similarities between a pregnant horn of a bicornuate uterus and an ectopic pregnancy [3].

The most feared complication of a pregnancy in a bicornuate uterus is the uterine rupture. The resulting blood loss can be the cause of death for both the fetus and the expectant mother [1]. A pregnancy implanted in a well-developed horn may continue without complications, but a pregnancy in the rudimentary horn is considered higher risk and needs frequent examination. The incidence rate of pregnancy in the rudimentary horn has been calculated to be 1 in 400,000 [4].

We present this case of an interstitial pregnancy in a bicornuate uterus, presenting as an ectopic pregnancy, and its subsequent management.

## Case presentation

The patient, 43 years old, G3P3, presented at the emergency department of the hospital with acute pain in the right iliac fossa (RIF) and blood loss from the vagina. She reported the beginning of the pain approximately 12 hours prior and steadily increasing. Gastrointestinal symptoms were absent. The patient’s obstetrical history included two caesarean sections, the last one six years ago. Her medical history included hypothyroidism for which T4 100 mg was being administered.

**Figure 1:**
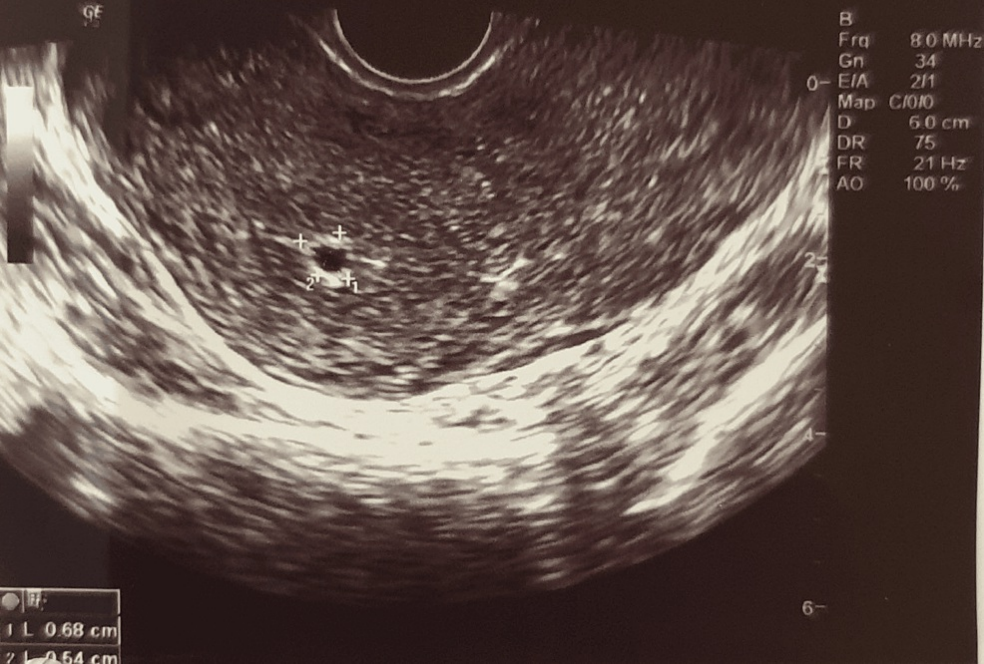
Gestation sac sized 0.68 x 0.54 cm

The vital signs of the patient were evaluated and found normal (blood pressure 134/73 mmHg, heart rate 80 bpm). Vaginal examination showed blood clots in the vagina, as well as minimal bleeding originating from the cervix. On palpation, the patient reported pain in the RIF and right vaginal wall. Blood tests were performed, showing 31.8 hematocrit and 1025.14 b-hCG. Transvaginal ultrasound scan revealed a bicornual uterus, which was not mentioned by the patient during the recording of the patient's obstetrical history (right uterine horn 5.89 x 3.69 x 4.10 cm, left uterine horn 5.91 x 3.70 x 3.78). At the bottom of the right horn, a cystic formation with signs of a gestation sac, sized 0.68 x 0.54 cm, was detected (Figure 1).

The patient was admitted to the hospital as an emergent case with the diagnosis of incomplete abortion. Dilation and curettage was planned. The endometrium was scraped in all directions and the operation was completed with no complications.

Post-operatively, the patient was examined via blood tests two days later. The haematocrit levels were minimally lowered and the patient was clinically stable, however, b-hCG did not show considerable change. A transvaginal ultrasound scan was performed to examine the patient. The scan showed the gestation sac in the same position as the last examination.

An exploratory laparoscopy was planned and performed the next day. Due to multiple adhesions, the operation was modified to an exploratory laparotomy. A Pfannenstiel incision was performed. Entering the peritoneal cavity, an interstitial ectopic pregnancy was detected in the right horn of the bicornuate uterus. The gestation sac was excised and cornuostomy was performed without intraoperative complications.

**Figure 2:**
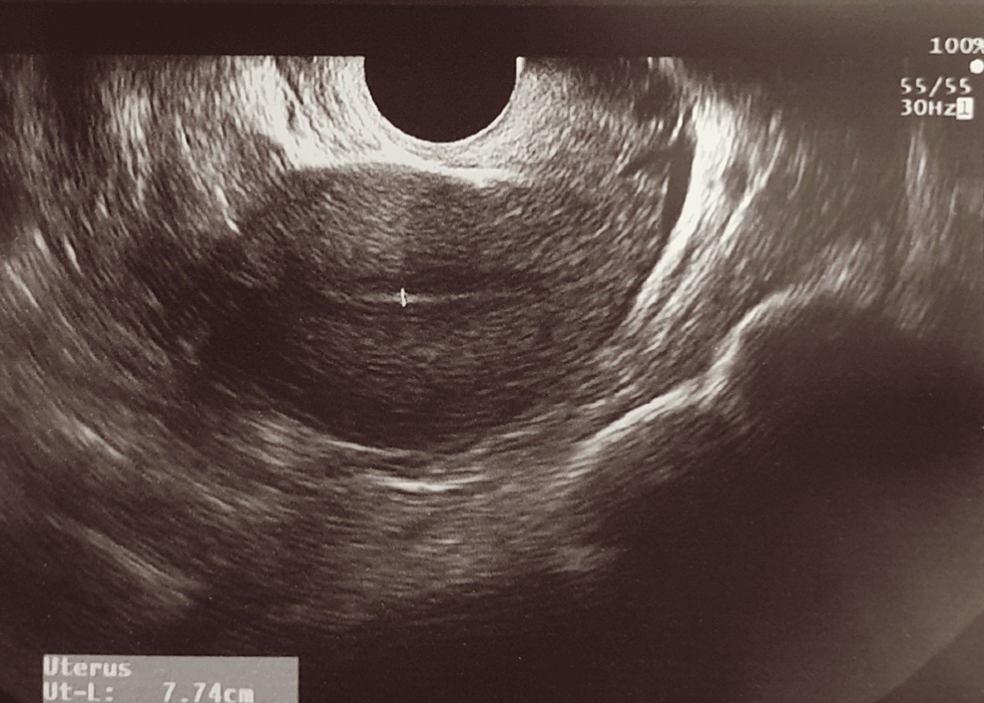
Ultrasound re-examination

Post-operatively, the patient was followed up with blood tests. B-hCG showed a rapid decrease in levels and the haematocrit had only minimal change. An ultrasound scan two days later showed the absence of the previously seen gestation sac (Figure 2). There were no complications post-operatively. The patient was discharged four days later.

The follow-up examination of the patient was performed a month later. The histology report presented an ectopic gestational sac with embryonic tissue. The patient was examined with an ultrasound scan and did not show signs of uterine rupture or free blood in the peritoneal cavity.

## Discussion

A common complication of the first trimester is the ectopic pregnancy. It is the cause of 9% to 13% of deaths during pregnancy worldwide, earning its position as a major threat to the health of expectant mothers. The most common implantation site of an ectopic pregnancy is the ampulla of the fallopian tube [5].

The mechanism of the establishment of an ectopic pregnancy is not understood, as of yet. Current theories point towards impaired tubular motility, ciliary dysfunction, functional tubal obstruction and molecular chemotactic factors encouraging implantation in the fallopian tube [6].

Symptoms of an ectopic pregnancy include pain in the lower abdominal region, hemorrhage originating from the vagina or secondary amenorrhea. Misdiagnosis can happen in the case of an intrauterine pregnancy or an early miscarriage [6].

Interstitial pregnancy is a less frequently encountered version of ectopic pregnancy. The fertilized ovum is implanted in the part of the fallopian tube close to the uterine body. A common outcome of this is rupture of the uterus and death due to massive hemorrhage. The interstitial part of the tube is able to enlarge to a greater extent before it breaks due to its thick walls. It is imperative to confirm such cases in a timely manner [7].

Transvaginal ultrasound is a powerful aid in the diagnosis of such cases. Common signs looked for are a gestation sac residing 1 cm away at least from the lateral edge of the cavity of the uterus, the absence of a chorionic sac in the cavity of the uterus and a layer of myometrium around the sac about 5 mm [7]. Characteristic is the interstitial line, which presents as an echogenic line linking the implantation site with the endometrial cavity [8].

Treatment is performed either pharmaceutically, with systemic methotrexate, or by surgical techniques, cornual resection and cornuostomy, and, in more radical cases, salpingectomy and hysterectomy [7]. In our case, after the failed attempt at dilation and curettage, an exploratory laparotomy was performed. The intact chorionic sac was detected on the right horn of the uterus and cornuostomy was performed. The patient was stable post-operatively, with no complications.

## Conclusions

Ectopic pregnancy is a serious condition that poses fatal consequences. When it coincides with rare congenital abnormalities, such as a bicornuate uterus, a necessity is created to have the best diagnostic tools at the disposal of the medical practitioners. Ultrasound scan is an important tool for recognizing the pathology of the patient that can aid practitioners in choosing the best therapeutic approach.

## References

[1] Tochie JN, Tcheunkam LW, Tchakounté C, Fobellah NN, Cumber SN (2020). First-trimester rupture of a gravid bicornuate uterus after prior vaginal deliveries, simulating a ruptured ectopic pregnancy: a case report. J Surg Case Rep.

[2] Tabatabaei F, Mohammadi Youshanloie M (2021). Successful delivery after uterine rupture with pervious open Strassman metroplasty for a bicornuate uterus in a twin pregnancy. Iran J Med Sci.

[3] Ngichabe S, Sura M (2017). Placenta percreta in a gravid bicornuate unicollis uterus. Case Rep Obstet Gynecol.

[4] Singh N, Singh U, Verma ML (2013). Ruptured bicornuate uterus mimicking ectopic pregnancy: a case report. J Obstet Gynaecol Res.

[5] Gari R, Abdulgader R, Abdulqader O (2020). A live 13 weeks ruptured ectopic pregnancy: a case report. Cureus.

[6] Taran FA, Kagan KO, Hübner M, Hoopmann M, Wallwiener D, Brucker S (2015). The diagnosis and treatment of ectopic pregnancy. Dtsch Arztebl Int.

[7] Di Tizio L, Spina MR, Gustapane S, D'Antonio F, Liberati M (2018). Interstitial pregnancy: from medical to surgical approach-Report of three cases. Case Rep Obstet Gynecol.

[8] Sargin MA, Tug N, Ayas S, Yassa M (2015). Is interstitial pregnancy clinically different from cornual pregnancy? A case report. J Clin Diagn Res.

